# From Admission to Discharge: Predicting National Institutes of Health Stroke Scale Progression in Stroke Patients Using Biomarkers and Explainable Machine Learning

**DOI:** 10.3390/jpm13091375

**Published:** 2023-09-14

**Authors:** Aimilios Gkantzios, Christos Kokkotis, Dimitrios Tsiptsios, Serafeim Moustakidis, Elena Gkartzonika, Theodoros Avramidis, Gregory Tripsianis, Ioannis Iliopoulos, Nikolaos Aggelousis, Konstantinos Vadikolias

**Affiliations:** 1 Department of Neurology, Democritus University of Thrace, 68100 Alexandroupolis, Greece; tsiptsios.dimitrios@yahoo.gr (D.T.); iiliop@hotmail.com (I.I.); vadikosm@yahoo.com (K.V.); 2 Department of Neurology, Korgialeneio—Benakeio “Hellenic Red Cross” General Hospital of Athens, 11526 Athens, Greece; avramidis@gmail.com; 3 Department of Physical Education and Sport Science, Democritus University of Thrace, 69100 Komotini, Greece; ckokkoti@affil.duth.gr (C.K.); s.moustakidis@aideas.eu (S.M.); nagelous@phyed.duth.gr (N.A.); 4 School of Philosophy, University of Ioannina, 45110 Ioannina, Greece; gkartzonika@yahoo.com; 5 Laboratory of Medical Statistics, Democritus University of Thrace, 68100 Alexandroupolis, Greece; gtryps@med.duth.gr

**Keywords:** stroke, biomarkers, severity, artificial intelligence, prognosis, interpretation, NIHSS

## Abstract

As a result of social progress and improved living conditions, which have contributed to a prolonged life expectancy, the prevalence of strokes has increased and has become a significant phenomenon. Despite the available stroke treatment options, patients frequently suffer from significant disability after a stroke. Initial stroke severity is a significant predictor of functional dependence and mortality following an acute stroke. The current study aims to collect and analyze data from the hyperacute and acute phases of stroke, as well as from the medical history of the patients, in order to develop an explainable machine learning model for predicting stroke-related neurological deficits at discharge, as measured by the National Institutes of Health Stroke Scale (NIHSS). More specifically, we approached the data as a binary task problem: improvement of NIHSS progression vs. worsening of NIHSS progression at discharge, using baseline data within the first 72 h. For feature selection, a genetic algorithm was applied. Using various classifiers, we found that the best scores were achieved from the Random Forest (RF) classifier at the 15 most informative biomarkers and parameters for the binary task of the prediction of NIHSS score progression. RF achieved 91.13% accuracy, 91.13% recall, 90.89% precision, 91.00% f1-score, 8.87% FN_rate_ and 4.59% FP_rate_. Those biomarkers are: age, gender, NIHSS upon admission, intubation, history of hypertension and smoking, the initial diagnosis of hypertension, diabetes, dyslipidemia and atrial fibrillation, high-density lipoprotein (HDL) levels, stroke localization, systolic blood pressure levels, as well as erythrocyte sedimentation rate (ESR) levels upon admission and the onset of respiratory infection. The SHapley Additive exPlanations (SHAP) model interpreted the impact of the selected features on the model output. Our findings suggest that the aforementioned variables may play a significant role in determining stroke patients’ NIHSS progression from the time of admission until their discharge.

## 1. Introduction 

As a consequence of social progress and improved living conditions, which have contributed to a longer life expectancy, the prevalence of strokes is a substantially established phenomenon. Furthermore, contemporary medical advancements have provided us with an expanded array of diagnostic techniques for the identification of stroke, resulting in an increased rate of stroke diagnoses. Despite the availability of numerous treatment options for stroke, it is common for stroke survivors to experience significant disability [1]. The severity of the initial stroke is a significant predictor of functional dependence and mortality following an acute stroke [2,3]. The assessment of such a prognosis is essential for the objective and precise evaluation of the disease severity in individuals with stroke [4]. Research has provided evidence that the establishment of regional and national stroke systems of care, in conjunction with initiatives aimed at enhancing quality, has a notable effect in diminishing stroke-related morbidity and enhancing patient outcomes [5,6,7]. Therefore, there is an ongoing need to improve our understanding of the prognosis and associated challenges. There are numerous data points regarding the variables influencing the prognosis, the severity, and the functional outcomes of stroke. The variables under consideration herein encompass the simultaneous factors of the genetic and demographic features of the patients, along with the data collected during the acute phase of the stroke and its related components [8].

In terms of stroke management as a whole, there have been no significant developments related to the acute phase treatment. We have a greater understanding of stroke causation than of stroke recovery. Consequently, pathophysiological mechanisms must be understood in greater depth for the rehabilitation to be more effective. Rehabilitation assists individuals in recovering their independence, abilities, and quality of life. Given the expanding availability of rehabilitation therapies, the absence of unanimity among metrics poses a hindrance to effectively harnessing clinical results and establishing a satisfactory degree of evidence for interventions. Therefore, a precise and comprehensive evaluation is required to assess patient recovery factors and to guide clinical treatment decisions [9,10,11].

The utilization of blood biomarkers has become increasingly recognized as a powerful diagnostic tool and an innovative method for accurately predicting functional outcomes subsequent to a stroke [12]. As a result, numerous risk-prediction models have been established with the aim of discerning functional outcomes during the initial stage of a stroke. The majority of these models form their prognostic assessments based on similar input criteria, such as age, initial stroke severity, and comorbidities. Nevertheless, the utilization of these prediction models in clinical practice has been limited, potentially as a result of challenges related to implementation [13,14,15,16]. Hence, the most challenging aspect of stroke rehabilitation research lies in the optimization of rehabilitation regimens by early diagnosis [17,18]. An accurate prognosis can be beneficial in the context of stroke discharge planning and individualized rehabilitation. Nevertheless, the task of forecasting patients’ outcomes, particularly in the early stages following admission, poses significant challenges [19,20,21,22].

Machine learning (ML) has been extensively utilized in various scientific disciplines, such as medicine, to address complex problems [23,24,25,26,27]. It is a subfield of artificial intelligence that involves the development of algorithms and models that utilize data patterns in order to predict forthcoming data. Computers possess the capability to efficiently and expeditiously handle extensive volumes of data, rendering them valuable tools for use. The concept of leveraging machine learning to expedite advancements in the field of healthcare through the automation of mundane chores and the augmentation of clinical decision-making holds significant appeal in contemporary times. The development of machine-learned solutions requires careful consideration of challenges such as inadequate data classification and gathering methods, as well as the intricate nature of clinical choices and procedures. ML could improve healthcare delivery. To genuinely assist patients, however, the development and implementation of new technologies must be methodical, inclusive, collaborative, and iterative [8,28,29].

To reduce the risk of strokes, scientists construct models that accurately foresee their occurrence. Artificial intelligence (AI) has played a significant role in disease prevention [30,31,32] within this framework and since its implementation. A growing number of studies [33,34,35] have demonstrated that ML methodology is more accurate at predicting the outcomes of strokes than statistical methods or scoring systems. ML and deep learning provide data-driven predictions of clinically significant outcomes for stroke patients using a vast array of structured data [16,36,37].

The primary objective of this study is to collect and analyze information regarding the hyperacute and acute stages of stroke, as well as the patient’s medical history. Using the National Institutes of Health Stroke Scale (NIHSS), this data analysis aims to develop a machine learning model capable of predicting the progression of clinical stroke severity from admission to discharge. The NIHSS quantifies stroke-related neurological impairments. It is designed to be administered in under 10 min by doctors, nurses, or therapists. It is a 15-item scale that assesses the effects of a stroke on consciousness, language, neglect, visual-field loss, extraocular movement, motor strength, ataxia, dysarthria, and sensory loss [38]. Each object is graded on a scale ranging from 3 to 5 points. Higher scores indicate a more severe condition. In the context of clinical practice, the utilization of this method enables the assessment and recording of the neurological status of individuals experiencing acute stroke. This facilitates the identification of the most suitable course of treatment and promotes the establishment of a consistent means of communication among healthcare practitioners [39]. The NIHSS has been shown to be a reliable predictor of both immediate and long-term prognoses in individuals who have experienced a stroke [40,41]. The NIHSS can be administered rapidly and is a reproducible and efficient screening method. As a method for evaluating patient progress over time, it has been incorporated into the clinical practice of a number of institutions [40,42].

Explainable artificial intelligence (AI) provides visible and interpretable explanations for its predictions and decisions. The goal of the study at hand is to properly predict stroke severity at discharge and find variables from the patient’s medical history and admission biomarkers and parameters that affect the NIHSS score at discharge. By tailoring rehabilitation strategies to each patient’s needs, this information can improve patient outcomes. Explainable AI helps healthcare personnel comprehend and trust the model’s predictions and conclusions, improving patient care.

## 2. Material and Methods 

### 2.1. Participants

From July 2017 to June 2018, a total of 413 patients were hospitalized with acute stroke at Korgialeneio–Benakeio “Hellenic Red Cross” General Hospital in Athens. The hospital’s Scientific Council authorized the study (protocol number 6673/08-03-2018). All patients were retrospectively observed until discharge. At both admittance and discharge, the National Institutes of Health Stroke Scale (NIHSS) was used to evaluate the severity of the stroke. We selected a sample population that consisted of patients over the age of 18 with an ischemic or hemorrhagic stroke and no prior functional deficits (mRS before stroke = 0).

### 2.2. Data Description 

This study involved the collection of patient data encompassing a comprehensive set of 32 separate factors or variables. The said parameters included demographic information such as age and gender, as well as stroke type (either ischemic or hemorrhagic) and admission levels of systolic blood pressure, glucose, CRP, and ESR. Age was categorized into the following 8 levels: <30 years, 30–39, 40–49, 50–59, 60–69, 70–79, 80–89, and >90 years. Additionally, we collected data pertaining to the patient’s medical background, encompassing conditions such as hypertension, smoking, diabetes, dyslipidemia, atrial fibrillation, previous stroke, prior myocardial infarction or coronary heart disease, history of heart failure, history of mechanical or bioprosthetic heart valve, history of alcoholism, history of antiplatelet drug usage and history of anticoagulant drug usage. Furthermore, we collected data pertaining to the first 72 h after the stroke occurrence. The latter included the occurrence of intubation, the initial diagnosis of hypertension, diabetes, dyslipidemia, and atrial fibrillation, as well as measurements of total cholesterol, low-density lipoprotein (LDL), HDL, low triiodothyronine (T3) thyroid hormone levels, and the presence or absence of respiratory infection. Moreover, stroke localization was documented concurrently, taking into account the blood supply and hemisphere stroke localization as determined using CT or MRI imaging of the brain. Lastly, we evaluated the severity of each patient’s stroke using the NIHSS at admission and discharge.

### 2.3. Problem Definition 

This study aims to accurately predict the severity of a stroke at the time of discharge by identifying the features of a patient’s medical history and admission biomarkers and parameters that influence the progression of the NIHSS score from admission to the time of discharge. Consequently, we formulated our primary inquiry as a binary question to ascertain the factors that influence the progression of the NIHSS within the designated time intervals. Thus, the aforementioned content was succinctly outlined in the subsequent proposal: Improvement of NIHSS progression vs. worsening of NIHSS progression at discharge using baseline data within the first 72 h.

*Input:* Our dataset included baseline data from the admission and within 72 h, as well as from the medical history of the patients (31 parameters). 

Class 1 (Improvement of NIHSS progression): This class contains post-stroke patients (n = 364) with an improvement in NIHSS progression upon discharge.

Class 2 (Worsening of NIHSS progression): Class 2 includes post-stroke patients (n = 49) with worsening of NIHSS progression upon discharge.

**Figure 1:**
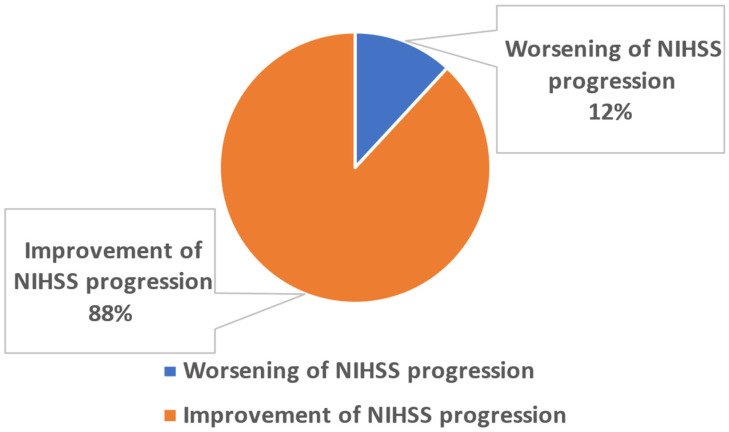
Grouping of the employed post-stroke patients.

*Output:* Classification outputs 0 and 1 corresponded to assignments to classes 1 and 2 (Figure 1), respectively. The NIHSS progression was calculated by Equation (1).

(1) $$dx_{i}^{k}=x_{i}^{k}-x_{i}^{0}\;$$

where, $x_{i}^{k}$ and $x_{i}^{0}$ are the NIHSS scores, which are measured at the visit *k* (at discharge) and the baseline, and *I* is the index of the employed post-stroke patients.

### 2.4. Proposed Methodology 

**Figure 2:**
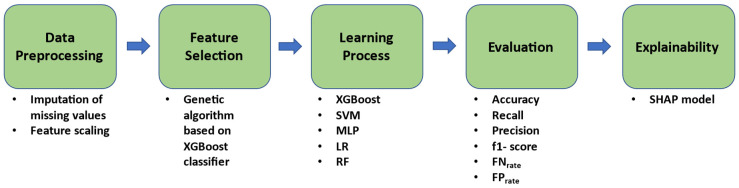
Workflow of the proposed methodology.

A hierarchical XAI machine learning pipeline (Figure 2) was created by our team in order to discover robust biomarkers and parameters that could predict the severity of outcome as determined by the progression of NIHSS score at discharge. The proposed methodology consisted of five steps, namely, data pre-processing, feature selection (FS), learning process, evaluation, and explainability. 

#### 2.4.1. Pre-Processing

In order to deal with missing data, the mode imputation strategy was used. This strategy is a technique used to handle missing data in a dataset. It involves replacing missing values with the most frequently occurring value in the dataset. This technique is particularly useful when the missing data are not random, as it can help to preserve the underlying structure of the data. Furthermore, in order to avoid issues such as overfitting or underfitting, the StandardScaler library (https://scikit-learn.org/, accessed on 10 March 2023) was employed. StandardScaler is a preprocessing library used to standardize the features of a dataset by centering and scaling the data to unit variance. This can help to ensure that the data are in a common scale and that the features have similar ranges of values.

#### 2.4.2. Feature Selection 

In our study, we employed an evolutionary-based strategy for feature selection [43]. Known for its proficiency in implementing search methodologies, a genetic algorithm (GA) was paired with a 5-fold cross-validation strategy. Our objective was to identify the most optimal feature subset that would enhance the performance of our classifier, which in this context was based on the XGBoost model. The GA iteratively generated candidate feature subsets of varying dimensionality. These subsets underwent evolutionary processes such as selection, crossover rate 0.8, population size 50, and mutation rate 0.1. The efficacy of each subset was assessed using a fitness function, determined by the training classification performance (5-fold CV) measured with the ROC AUC score. Feature subsets that showcased the highest fitness values were then chosen as the foundation for the subsequent generation. This evolution ran across a specified number of generations (n = 100) or until a defined stopping criterion was met. The culmination was the feature subset that exhibited the highest fitness, which ultimately consisted of 15 features. While the GA feature selection method was adept at managing datasets rich in features and its versatility with a variety of classifiers was evident, its computational intensity was notable. There was potential for prolonged generational cycles before arriving at an optimal solution, especially when a powerful model like XGBoost was used as the baseline criterion.

#### 2.4.3. Learning and Validation Strategy 

For the learning process, we employed five well-known classifiers such as Logistic Regression (LR) [44], XGBoost (eXtreme Gradient Boosting) [45], Random Forest (RF) [46], Multilayer Perceptron (MLP) [47] and Support Vector Machine (SVM) [48]. LR is a statistical method for analyzing a dataset in which there are one or more independent variables that determine an outcome. It is a linear classifier, which means that it can predict the probability of a certain class, and it is fast and easy to interpret. XGBoost is an open-source software library that provides an efficient implementation of gradient boosting. It is a powerful and efficient algorithm that is particularly good at handling large datasets and high-dimensional feature spaces. XGBoost is an ensemble method, which means that it combines multiple decision trees to make a final prediction. RF is an ensemble learning method that combines multiple decision trees to improve prediction accuracy and reduce overfitting. MLP is a type of artificial neural network (ANN) that can be used for supervised learning tasks such as classification and regression. It is a fully connected feedforward neural network that consists of multiple layers of artificial neurons. MLP is trained using backpropagation, which is a supervised learning algorithm that adjusts the weights of the network to minimize the difference between the predicted and actual output. It is a non-linear classifier that is suitable for non-linearly separable data. SVM is a supervised machine learning algorithm that can be used for classification and regression tasks. SVM tries to find the hyperplane that maximizes the margin between the two classes in a feature space. It also has a kernel trick that can transform the data into a higher dimensional space that allows for non-linearly separable data to be separated in this space.

A 70%/30% training/testing validation strategy was used to evaluate the performance of a model on unseen data, while internal 10-fold cross-validation was used to tune the hyperparameters (Table 1) of the models in order to improve their performance. Furthermore, internal oversampling was performed in the training set. Internal oversampling is a strategy to tackle this problem by artificially increasing the representation of the minority class within the training data. This strategy helps to prevent the model from becoming biased toward the majority class and provides the model with a more balanced perspective, helping it learn patterns including the minority class. As validation metrics, we used accuracy, recall, precision, f1-score, FN_rate_ and FP_rate_, Matthew’s correlation coefficient, and precision-recall curve [49].

#### 2.4.4. Explainability 

The SHAP (SHapley Additive exPlanations) model is based on the concept of Shapley values from cooperative game theory [50,51]. The SHAP library provides a powerful tool for understanding how a machine learning model arrived at its predictions and identifying the features that were most important in the decision-making process. By using the concept of Shapley values, SHAP assigns feature importance values that are more accurate than other methods, and it can be used to interpret any model that outputs a scalar value.

## 3. Results 

This section presents the testing results of the classifiers that were trained using the 15 most informative biomarkers. Additionally, this section provides the 15 most informative biomarkers and the interpretability of the model output from the best ML model.

### 3.1. Prediction Performance 

Table 2 demonstrates the performance metrics of the employed ML classifiers. The best scores were achieved from the RF classifier at the 15 most informative biomarkers and parameters for the binary task of the prediction of NIHSS score progression. RF achieved 91.13% accuracy, 91.13% recall, 90.89% precision, 91.00% f1-score, 8.87% FN_rate_ and 4.59% FP_rate_. The aforementioned scores of the RF classifier were achieved with Gini as the criterion, minimum samples leaf = 1 and minimum samples split = 1. On the contrary, the lowest scores in the same task were achieved by the SVM (C = 7, kernel = poly and gamma = scale). SVM achieved 83.07% accuracy, 83.07% recall, 85.38% precision, 84.08% f1-score, 16.94% FN_rate_ and 11.92% FP_rate_. Furthermore, the MLP classifier achieved 87.90% with activation = ReLU, alpha = 0.0001, hidden_layer_sizes = (10, 20, 50), learning rate = adaptive, and solver = Adam; the XGBoost classifier achieved 86.29% with gamma = 0, maximum depth = 4, and minimum child weight= 1; and the LR classifier achieved 84.68% accuracy with C = 1 and penalty = l2.

**Figure 3:**
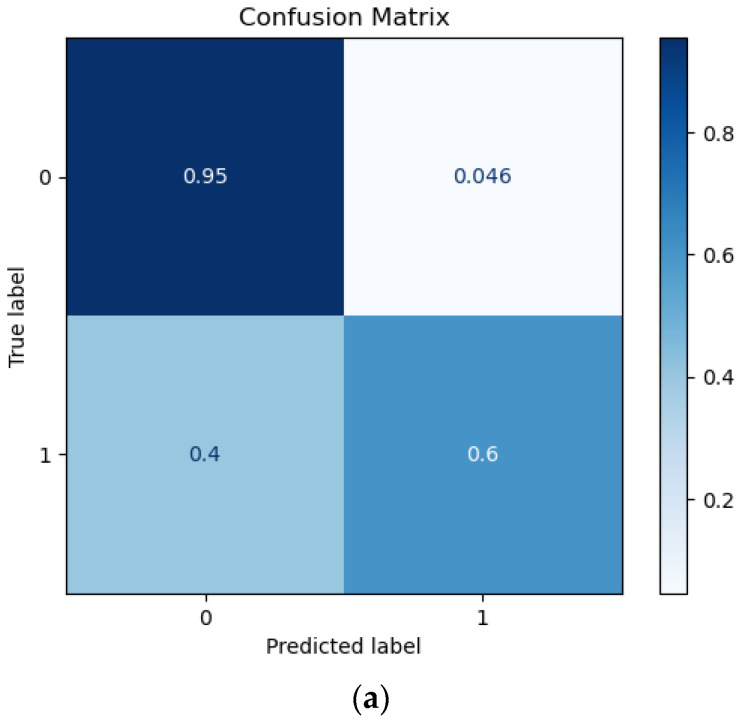

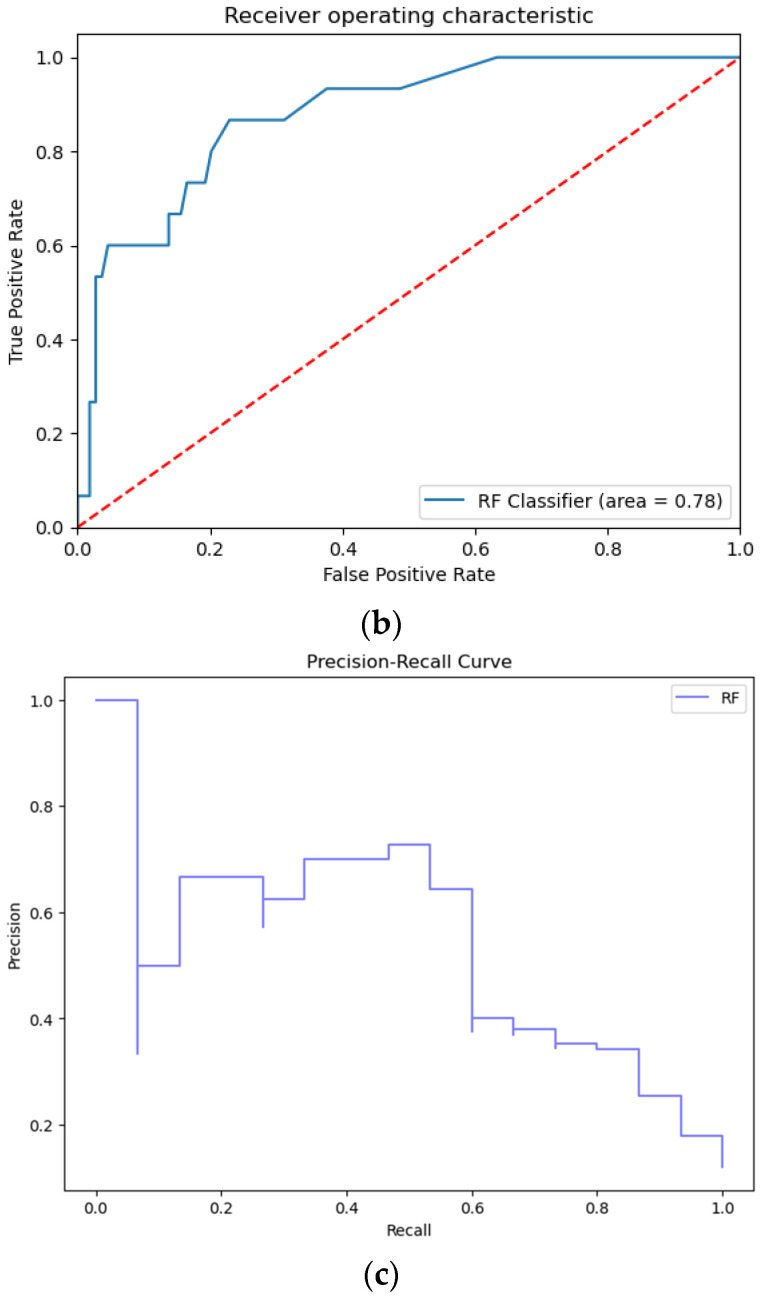
(**a**) Confusion matrix, (**b**) the receiver operating characteristic, and (**c**) the precision-recall curve of the RF classifier.

Furthermore, the confusion matrix and the receiver operating characteristic (AUC = 0.78) and the precision-recall curve for the best ML model (RF classifier) are presented below (Figure 3). Furthermore, the RF classifier achieved a 0.57 Matthew’s correlation coefficient score.

### 3.2. Selected Features

Table 3 displays the 15 most significant biomarkers and parameters that were determined through the use of the genetic algorithm as an FS technique in predicting the NIHSS score progression at discharge in a binary problem. 

### 3.3. Explainability Analysis 

**Figure 4:**
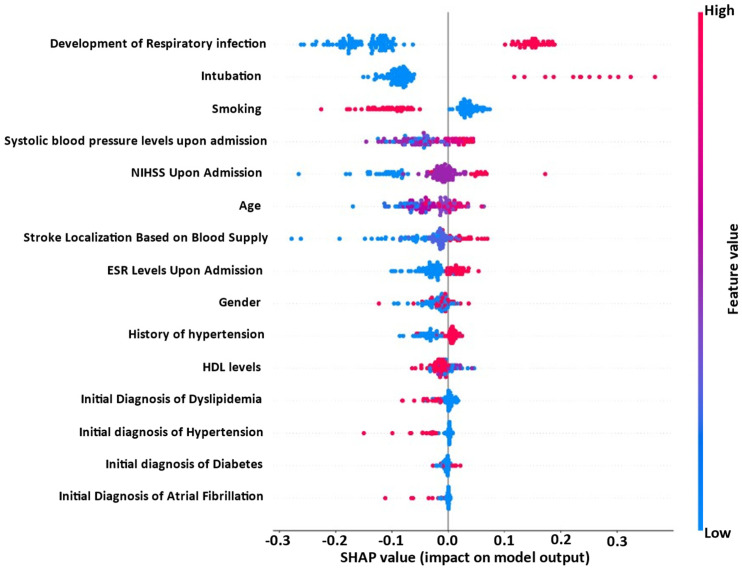
Biomarker and parameter impact on RF model output for the prediction of NIHSS progression. The distribution of the impact of a biomarker and parameter on the model output across test instances.

Figure 4 demonstrates the impact of the 15 most significant biomarkers and parameters on the best performing ML model’s (RF) output. Figure 4 is displayed with the most influential biomarkers and parameters in descending order, shown in a top-down perspective. The color indicates the level of the risk factor for each individual observation, with red indicating a high value and blue indicating a low value. The development of respiratory infection in post-stroke patients is a significant risk factor that contributes to the worsening of NIHSS score progression. In more detail, the occurrence of respiratory infection leads to an increase in the model output, indicating a higher likelihood of post-stroke patients experiencing a deteriorating NIHSS progression. Therefore, there is a clear association between the development of respiratory infection and unfavorable NIHSS score progression. Furthermore, high values of systolic blood pressure levels upon admission, NIHSS upon admission, age, ESR levels upon admission, and the history of hypertension, intubation, and stroke localization have a positive impact on the worsening of NIHSS score progression. On the contrary, smoking status and HDL levels are negatively correlated with the worsening of NIHSS score progression. 

## 4. Discussion 

Within this section we will present the findings of our investigation. Considering that, it must be mentioned that, to date, researchers have evaluated each of the aforementioned factors separately but not in conjunction with one another and it has not been determined which factors influence the progression of the clinical severity of stroke, as measured by the NIHSS scale. Thus, it is necessary to investigate which factors influence this progression. What follows our findings is a brief literature review of the studies to date that have used machine learning in their analysis of similar topics, using the NIHSS as the primary scale for assessing clinical severity, as well as a brief assessment of each parameter that our research indicated influences the clinical outcome of patients, as indicated by changes in the NIHSS scale.

The clinical outcome of a stroke is variable and dependent on numerous variables. Due to advances in medical knowledge and technology, the objective assessment of disease severity in stroke patients can provide a premise for prognostication and medical decision-making. Having identified and utilized the aforementioned 31 parameters upon admission, in the first 72 h and from the medical history of stroke patients, we attempted to develop an explainable machine learning model for the prediction of NIHSS progression during the hospitalization of stroke patients, specifically from admission to discharge. Under this lens, in our study, a hierarchical XAI machine learning pipeline was implemented to identify those biomarkers and parameters that are capable of predicting the severity of outcome. The methodology proposed comprises five steps, namely, data preprocessing, feature selection, learning process, evaluation, and explainability. The Random Forest (RF) classifier achieved superior performance in predicting the evolution of NIHSS scores for the binary task, utilizing the top 15 most useful biomarkers and covariates. The Random Forest (RF) model demonstrated a high level of performance, with an accuracy of 91.13%, recall of 91.13%, precision of 90.89%, and an f1-score of 91.00%. Additionally, the false negative rate (FNrate) was found to be 8.87% and the false positive rate (FPrate) was 4.59%. Among the 15 features that exhibited the highest degree of informativeness, several factors demonstrated a positive correlation with the worsening of clinical severity of stroke, as indicated by NIHSS progression. These factors included the development of respiratory infection, elevated systolic blood pressure levels at admission, NIHSS score at admission, advanced age, elevated ESR levels at admission, and a history of hypertension, intubation, and stroke localization. Conversely, smoking status and HDL levels displayed a negative association with the worsening of clinical severity, as depicted by NIHSS progression. The five remaining criteria, namely, gender, initial diagnosis of hypertension, diabetes, dyslipidemia, and atrial fibrillation (AF), appear to have a neutral impact on the progression of the NIHSS. 

To date, the number of studies employing machine learning in order to analyze data with respect to the prediction of the evolution of clinical severity as depicted by the NIHSS remains limited. More specifically, Lai et al. [52] conducted a study in which they utilized a pretrained VGG-16 convolutional neural network (CNN) to predict the mRS and NIHSS scores after discharge, specifically within a time frame of 28 ± 3 days. A total of 44 post-stroke patients were included in the study during the acute phase. The predictive accuracy for the National Institutes of NIHSS was found to be 92.7%, while the mRS had a predictive accuracy of 93.2%. Moreover, Rajashekar et al. [53] introduced a novel approach involving nested regression models in order to forecast the 30-day NIHSS score. The researchers utilized imaging data and quantifiable clinical data, which were acquired during a time frame of up to six hours following the occurrence of a stroke. This study involved the development of an SVM regression model that incorporated both non-modifiable and modifiable risk factors. Additionally, two nested SVM regression models were created, which combined image-based and clinical features. The said models differed in terms of the feature selection (FS) approach put to use. The initial approach utilized the relief FS technique, while the subsequent approach had recourse to the lesion-symptom mapping technique. The Mrelief model demonstrated the highest performance results, with a mean absolute error (MAE) of 3.55, root mean square error (RMSE) of 4.34, and R2 of 0.43.

Having presented our method and the current literature on the subject, we shall further present a concise literature review of each parameter our research showcases that plays a role in the clinical progression of patients, as indicated by NIHSS changes. In light of the fact that researchers have evaluated each of the aforementioned factors individually but not in combination and it has not been determined which factors influence the progression of clinical stroke severity as measured by the NIHSS scale, it is necessary to investigate which factors influence this progression. Τhe correlation between age, stroke risk, and clinical severity is well established. Of all strokes, 75% occur in individuals above 65 years of age, and the incidence of strokes in adults over 75 years of age is more than double that of those under 65 [54]. Approximately 50% of all strokes occur in individuals over the age of 75 and about 30% in those over the age of 85 [55,56]. Several subtypes of stroke, including atherosclerosis, atrial fibrillation, and small vessel disease, are strongly associated with age [57,58,59]. Additionally, elderly patients with ischemic stroke frequently have poorer outcomes compared to younger patients [60,61]. Also, substantially higher among the elderly were the discharged NIHSS scores, the mRS, and the length of hospital stay. These adverse results may be attributable to an increased severity of stroke, as measured by initial NIHSS scores, among the elderly [62,63].

In terms of presentation, severity, etiology, and prognosis, the influence of gender on acute stroke is becoming progressively apparent. There is a disproportionate burden of cerebrovascular disease among women, which is increasing the recognition of gender disparities in stroke [64]. Men have higher age-specific stroke rates. Nevertheless, due to women’s longer life expectancy and the significantly higher incidence of stroke at older ages, women experience more strokes than males overall. However, that fact partially explains the higher incidence of strokes among women [65]. Studies indicate that women who present with more severe neurologic impairments are less likely to receive acute stroke therapies, and they experience a worse functional prognosis after hospitalization [66,67,68]. To this end, Caso et al. [69] found that the admission NIHSS scores of women were higher than those of men. Statistically, women were more likely than men to encounter a cardioembolic stroke. Men had a higher incidence of lacunar and atherosclerotic strokes compared to women. Supporting the preceding research, Santalucia et al. [70] discovered in their study that women presented with more severe strokes at onset than men, as measured by the NIHSS, and that women are reported to experience more aphasic disorders, visual field disturbances, and dysphagia than men, while there have been no reported disparities in either motor or sensory function. Men are more likely than women to experience cerebellar and brainstem symptoms, as well as higher rates of posterior circulation syndromes.

Hypertension is the most prevalent modifiable risk factor associated with stroke [71]. Elevated initial blood pressure (BP) is a frequently seen prognostic factor in cases of acute ischemic (IS) and hemorrhagic stroke (HS), and it is associated with worse short- and long-term outcomes. Previous studies on stroke have identified relations between initial blood pressure (BP) and outcomes that follow a J- or U-shaped pattern [72]. High admission BP in IS enhances early neurological impairment and predicts poor 90-day outcomes, and high BP during admission increases hematoma extension risk and predicts poor clinical outcome and mortality in HS [73,74]. Finally, extremely high or low blood pressure at admission is linked to a more severe stroke, greater admission costs, and worse one-year outcomes [75,76].

Diabetes mellitus is a known risk factor for stroke and may be associated with a worse prognosis following a stroke. It is associated with numerous cardiovascular risk factors, including hypertension, hyperlipidemia, obesity, and insulin resistance, and it causes atherosclerotic alterations in various blood vessels. Specifically, aberrant glucose regulation, of which diabetes is a manifestation, is present in up to two-thirds of stroke patients and increases the risk of death or severe disability in diabetic patients. Previously undiagnosed diabetes and impaired glucose tolerance account for an additional 5% to 28% [77,78,79].

For both men and women, smoking is a well-established independent risk factor for all types of stroke. Smoking induces hypercoagulation, which regulates hematocrit and fibrin-rich clots, increases fibrinogen levels, and inhibits fibrinolysis. Furthermore, smoking is linked to a rise in plasma carbon monoxide concentration. Consequently, smoking increases the risk of stroke by three to four times, whereas exposure to environmental smoke increases the risk by 1.5 to two times. Nonetheless, the observation that smokers, particularly young adults, may have a better prognosis for stroke than nonsmokers gave rise to the term “smoking paradox.” The smoking paradox refers to the favorable prognosis of smokers with cardiovascular conditions during clinical treatment in comparison to nonsmokers, first observed in post myocardial infarction and later in ischemic stroke. Among stroke or heart disease patients undergoing intravenous thrombolysis with tPA (tissue-type plasminogen activator), smoking was found to improve functional outcome. Currently, the NIHSS upon admission has been acknowledged as a distinct indicator of functional recovery. However, the correlation between smoking status and outcome has yet to be demonstrated [80,81,82,83,84].

In the preponderance of observational studies, higher total cholesterol (TC) and LDL-C levels are associated with an increased risk of ischemic stroke [85,86,87]. Additionally, several observational studies found a correlation between lower TC and LDL-C levels and a higher risk of hemorrhagic stroke [88,89,90,91]. Research findings also indicate an inverse relationship between high-density lipoprotein cholesterol (HDL-C) and stroke incidence. The association between high-density lipoprotein (HDL) and cerebrovascular disease is believed to be dependent on HDL-C subfractions rather than overall HDL-C, as indicated by inconsistent research findings and the lack of impact of HDL-increasing drugs on the risk of ischemic stroke. Specifically, high-density lipoprotein (HDL) can be divided into two primary subfractions: HDL-C (HDL2), characterized by greater size and lower density, and HDL-C (HDL3), characterized by smaller size and higher density. The biological activity, biochemical properties, and vascular metabolism of the subfractions exhibit variations. HDL3 inhibits LDL oxidation and protects against atherosclerosis more than HDL2 through acting on the vascular endothelium. HDL subfractions altered carotid disease risk differently: HDL2 correlated with plaque thickness and HDL3 with plaque area [92,93,94,95,96]. Consequently, it is evident that there is a complex relationship between the lipid profile and stroke. It affects the risk of stroke and its individual parameters are associated differentially with ischemic and hemorrhagic stroke [97].

Frequently undetected, atrial fibrillation (AF) is a significant risk factor for stroke. It increases the risk of IS for individuals of all ages, but particularly the elderly. Patients with AF have more severe strokes (as assessed by the NIHSS score) and neurologic impairments than patients with ischemic stroke who do not have AF [98,99]. In patients with atrial fibrillation, ischemic strokes are likely to be severe or fatal. With oral anticoagulants, patients with atrial fibrillation can significantly reduce their risk of stroke [100]. AF detection and anticoagulation are necessary for preventing stroke. Only 50% of AF patients have symptoms. It is challenging to detect AF in individuals without symptoms. AF is identified in 7.8% to 36.2% of acute ischemic stroke patients after the index stroke. Nevertheless, research indicates that stroke severity and hospital outcomes are comparable for newly diagnosed and previously diagnosed AF patients. Although individuals with newly diagnosed AF after stroke and those with known AF prior to stroke had distinct baseline characteristics, the difference in stroke severity has not been sufficiently investigated [101,102,103,104,105,106].

Inflammation plays a significant role in the etiology of atherosclerosis. The erythrocyte sedimentation rate (ESR) is a valuable method for assessing disease activity in a variety of inflammatory and non-inflammatory conditions. The ESR is a parameter that measures the tendency of red blood cells (RBCs) to aggregate. It is frequently employed in routine analysis for the primary purpose of detecting hidden inflammation [107]. The occurrence of an ischemic stroke is associated with a significant disruption in hemorrhagic processes, which impairs blood flow by modifying plasma viscosity and erythrocyte aggregation. By impeding cerebral blood flow, these changes contribute to the progression of ischemia. It has been proposed to utilize erythrocyte aggregation as a potential indicator for identifying the presence of inflammation in stroke. Clinicians now indirectly evaluate this inflammatory state by measuring the ESR. There are studies that support the notion that the rise in ESR levels following a stroke in some patients should not be interpreted as an indication of inflammation. Others support that examining patients’ ESR levels on a regular basis can serve as an indicator of ongoing ischemic stroke events [108,109]. Nonetheless, it is essential to take into account a variety of factors, including hematological parameters and other robust markers of inflammation, such as C-reactive protein, fibrinogen, and triglycerides. In previous studies, an elevated ESR has been associated with a larger infarct size. Shortly after an acute ischemic stroke (AIS), the ESR values may indicate the presence of an acute phase response and the severity of localized brain injury. Through activation of pro-inflammatory and pro-thrombotic pathways, which result in the aggregation of erythrocytes, the acute phase response contributes to the exacerbation of tissue damage in response to tissue injury. Therefore, the ESR may be regarded as an indicator of the acute phase response, and its evaluation upon admission may serve as a prospective indicator of the severity of AIS. Given that the ESR is a non-specific indicator of inflammation that can be affected by a number of factors, it is imperative that the results be interpreted in conjunction with other pertinent clinical observations. Considering that stroke patients exhibit multiple risk factors for atherosclerosis, such as hypertension, diabetes mellitus, and smoking, it is plausible that they manifest a pre-existing state of elevated inflammation or blood clotting, which could potentially contribute, to some extent, to the increase in the ESR shortly after the stroke [110,111].

Approximately 30% of patients experience post-stroke infections during the initial days following the event, with pneumonia accounting for approximately one-third of these cases [112]. Stroke-associated pneumonia often occurs as a result of aspiration. Patients who are hospitalized and experiencing neurological damage often exhibit impaired swallowing reflexes, rendering them vulnerable to the development of aspiration [113,114]. There exists a correlation between the occurrence of an infection subsequent to a stroke and an increased likelihood of experiencing an unfavorable result or mortality. Pneumonia is a significant factor in premature mortality following a stroke, with an estimated 10% of fatalities occurring within 30 days after the stroke being directly linked to pneumonia [115,116,117,118]. The majority of the cases of pneumonia manifest within the initial 48 to 72 h following a stroke. Understanding the temporal pattern can help determine the most opportune timeframe for implementing antibiotic interventions aimed at preventing pneumonia in stroke patients [119,120,121].

Intubation for the purpose of mechanical ventilation (MV) is a commonly employed procedure in stroke patients as a result of impaired swallowing function and a compromised airway or respiratory system [122]. According to extensive population studies conducted across multiple centers, it has been observed that mechanical ventilation (MV) is necessary for approximately 10–15% of patients who are admitted to a hospital for acute stroke. The need for MV varies depending on the type of stroke, with subarachnoid hemorrhage (SAH) and intracranial hemorrhage (ICH) patients requiring MV at a rate that is three to four times higher than that of acute ischemic stroke (AIS) patients. Specifically, SAH and ICH patients require MV in approximately 29% and 30% of cases, respectively, while AIS patients require MV in approximately 8% of cases [123,124]. The primary determinant for the requirement of mechanical ventilation (MV) in stroke patients is likely the precise site of the stroke, rather than the exact type of cerebrovascular disease. Within this particular framework, the risk of respiratory failure is heightened as a consequence of the dysfunction of brain regions responsible for regulating consciousness levels (including the thalami, limbic system, and reticular formation in the brainstem), as well as those involved in controlling breathing (such as the respiratory centers located in the cortex, pons, and medulla) and swallowing (involving the medulla and brainstem connections). The prognosis of stroke patients who require mechanical ventilation is observed to be unfavorable, as indicated by hospital mortality rates ranging from 53% to 57% and 1-year mortality rates ranging from 60% to 92%. While the utilization of mechanical ventilation is often used as an indicator of the severity of a patient’s condition, it is important to note that the decision to perform endotracheal intubation may be influenced by the presence of potentially reversible conditions such as status epilepticus, pneumonia, sepsis, or hydrocephalus. These conditions have the potential to be resolved quickly and may lead to more positive outcomes for the patient [125,126,127,128].

The localization of stroke, which encompasses the detection of the affected hemisphere or blood supply, exhibits a multifaceted connection with the spatial distribution of the injury and its implications for clinical severity and functional recuperation. It is widely recognized that when a stroke occurs in the dominant hemisphere—typically the left hemisphere for most individuals, as opposed to the non-dominant or right hemisphere—it can result in specific clinical symptoms and deficits that depend on the specific regions that have been affected [129,130,131]. Several studies have also demonstrated that the participation of the right hemisphere is indicative of poorer functional results in individuals with stroke. Differentiation is of utmost importance in understanding the expected alterations in the link between lesion location, clinical impairments, recovery, and functional outcome, as these relationships are influenced by the specific site of the lesion. Conversely, there have been findings suggesting that there is no discernible impact of hemisphere lateralization on functional results. The impact of lesion location on the clinical prognosis of patients who have experienced a stroke is readily apparent. As a result, prediction models must incorporate this factor [132,133].

Our research is not devoid of limitations. Although the NIHSS scale is extensively utilized, its limitations persist, a fact that therefore has an impact on our research since it served as the primary instrument for assessing the clinical severity of stroke. More specifically, the consensus within the academic community is that the NIHSS is not a substitute for a comprehensive neurological assessment in terms of identifying the specific location of a lesion or assessing the consequences of modest impairments. The primary factor contributing to the issue is the lack of precision in the scale. The NIHSS has the potential to underestimate the severity of lesions in the posterior circulation, right hemisphere, and brainstem infarctions. There is currently no comprehensive assessment available for evaluating all cranial nerves. Due to its emphasis on gross motor skills and language-related activities, this particular approach exhibits a reduced vulnerability to domain modifications such as cognitive impairment, which may otherwise impact functional outcomes. The presence of an NIHSS abnormality does not always exclude the possibility of a stroke. Overall, there exists variability among institutions in terms of the methods employed for monitoring and recording neurologic function in hospitalized patients. Furthermore, given that the current study is retrospective, specifically referring to the database’s establishment prior to the widespread implementation of intravenous thrombolysis for patients with ischemic stroke and the advent of the mechanical thrombectomy period, it is imperative that our findings be validated by future research conducted on a prospective population. Although we are confident in the predictive capabilities of the current parameters for shaping the NIHSS progression from admission to discharge, we anticipate a decrease in the proportion of patients with severe or moderate stroke. This reduction is expected to lead to an improved functional recovery due to the implementation of intravenous thrombolysis in a subset of stroke patients. Furthermore, as far as our data analysis is concerned, imbalanced data required novel approaches to their management and the FS work we did; despite being time-consuming and requiring extensive fine-tuning to function properly, we believe that it was well served, within the framework of the following limitations. Finally, it is also important to acknowledge that the results presented in this study are specific to a single institution, namely, the Neurological Clinic. Although this clinic stands out from other neurological clinics in Greece due to the fact that it handles a substantial volume of stroke patients across all age groups, it is imperative to acknowledge that the sample under study reflects certain regional and socioeconomic characteristics.

The primary aim of this study was to utilize machine learning techniques in order to identify a comprehensive range of parameters that may have an impact on the progression of the NIHSS scale from patient admission to hospital discharge. Subsequent research endeavors will strive to undertake a more exhaustive examination of each of these attributes in correlation with both the clinical severity of stroke and the resulting functional outcome.

## 5. Conclusions

In summary, this research study identified 31 parameters at the time of admission, within the first 72 h, and from the medical history of stroke patients. The study’s objective was to develop an interpretable machine learning model that could predict the clinical severity of stroke, specifically measured by the progression of the NIHSS from admission to discharge. The RF classifier demonstrated superior performance in predicting the evolution of NIHSS scores by utilizing a set of 15 highly relevant biomarkers and characteristics. The Random Forest (RF) model demonstrated a high level of performance, achieving an accuracy of 91.13%. Additionally, the model exhibited a recall rate of 91.13%, a precision rate of 90.89%, and an f1-score of 91.00%. The false negative rate was observed to be 8.87%, while the false positive rate was found to be 4.59%. In contrast, the Support Vector Machine (SVM) exhibited the lowest results in the same challenge. The Support Vector Machine (SVM) model demonstrated an accuracy of 83.07%, recall of 83.07%, precision of 85.38%, f1-score of 84.08%, false negative rate of 16.94%, and false positive rate of 11.92%. 

To the best of our knowledge, the present study is an initial attempt to collect data during the early stage of stroke along with the identification of parameters from the medical records of these stroke patients. The data we collected were then subjected to analysis using machine learning techniques, with the aim of constructing a prognostic model that predicts the progression of clinical severity in stroke patients from the time of their admission to their discharge from the hospital, an endeavor that has the potential to enhance the timeliness, accuracy, and personalized nature of diagnosis and treatment for stroke patients. These data can ultimately be utilized to bolster the process of rehabilitation decision-making and enhance patient outcomes through the customization of rehabilitation regimens to suit the unique requirements and attributes of particular patients. 

## Figures and Tables

**Table 1:** Hyperparameters of the employed ML models.

Classifier	Hyperparameters
LR	penalty = l1, l2 C = 0, 1, 2, 4, 10
XGBoost	maximum depth: 1, 2, 3, 4, 5, 6, 7, 8, minimum child weight: 1, 3, 4, 5, 6, 8, gamma: 0, 0.4, 0.5, 0.6
RF	criterion: Gini, entropy, n estimators: 10, 15, 20, 25, 30, minimum_samples leaf: 1, 2, 3, minimum samples split: 3, 4, 5, 6, 7
MLP	hidden_layer_sizes: (2, 5, 10), (5, 10, 20), (10, 20, 50), activation: tanh, ReLU, solver: SGD, Adam, alpha: 0.0001, 0.05, learning rate: constant, adaptive
SVM	C: 0.001, 0.01, 0.1, 1, 2, 3, 4, 5, 6, 7, 8, 9, 10, kernel: linear, sigmoid, RBF, poly gamma: scale

**Table 2:** Metrics of the employed ML models.

Classifier	Accuracy (%)	Recall (%)	Precision (%)	f1-Score (%)	FN_rate_ (%)	FP_rate_ (%)
LR	84.68	84.68	92.24	86.81	6.67	16.51
XGBoost	86.29	86.29	87.48	86.82	13.71	9.17
**RF**	**91.13**	**91.13**	**90.89**	**91.00**	**8.87**	**4.59**
MLP	87.90	87.90	88.25	88.07	12.10	7.34
SVM	83.07	83.07	85.38	84.08	16.94	11.92

**Table 3:** Selected features based on genetic algorithm.

Features	Type of Data
Age	Categorical
Gender	Categorical
NIHSS upon admission	Categorical
Intubation	Categorical
History of hypertension	Categorical
Smoking	Categorical
Initial diagnosis of hypertension	Categorical
Initial diagnosis of diabetes	Categorical
Initial diagnosis of dyslipidemia	Categorical
HDL levels	Categorical
Initial diagnosis of atrial fibrillation	Categorical
Stroke localization based on blood supply	Categorical
Systolic blood pressure levels upon admission	Categorical
ESR levels upon admission	Categorical
Development of respiratory infection	Categorical

## Data Availability

The dataset generated and/or analyzed during the current study is not publicly available.
